# A robust and adaptive framework for interaction testing in quantitative traits between multiple genetic loci and exposure variables

**DOI:** 10.1371/journal.pgen.1010464

**Published:** 2022-11-16

**Authors:** Julian Hecker, Dmitry Prokopenko, Matthew Moll, Sanghun Lee, Wonji Kim, Dandi Qiao, Kirsten Voorhies, Woori Kim, Stijn Vansteelandt, Brian D. Hobbs, Michael H. Cho, Edwin K. Silverman, Sharon M. Lutz, Dawn L. DeMeo, Scott T. Weiss, Christoph Lange

**Affiliations:** 1 Channing Division of Network Medicine, Department of Medicine, Brigham and Women’s Hospital, Boston, Massachusetts, United States of America; 2 Harvard Medical School, Boston, Massachusetts, United States of America; 3 Genetics and Aging Unit and McCance Center for Brain Health, Department of Neurology, Massachusetts General Hospital, Boston, Massachusetts, United States of America; 4 Division of Pulmonary and Critical Care Medicine, Brigham and Women’s Hospital, Boston, Massachusetts, United States of America; 5 Department of Medical Consilience, Division of Medicine, Graduate School, Dankook University, Yongin, South Korea; 6 Department of Biostatistics, Harvard T.H. Chan School of Public Health, Boston, Massachusetts, United States of America; 7 Department of Population Medicine, PRecisiOn Medicine Translational Research (PROMoTeR) Center, Harvard Pilgrim Health Care, Boston, Massachusetts, United States of America; 8 Systems Biology and Computer Science Program, Ann Romney Center for Neurological Diseases, Department of Neurology, Brigham and Women’s Hospital, Boston, Massachusetts, United States of America; 9 Broad Institute of MIT and Harvard, Cambridge, Massachusetts, United States of America; 10 Department of Applied Mathematics, Computer Science and Statistics, Ghent University, Gent, Belgium; 11 Department of Medical Statistics, London School of Hygiene and Tropical Medicine, London, United Kingdom; Newcastle University, UNITED KINGDOM

## Abstract

The identification and understanding of gene-environment interactions can provide insights into the pathways and mechanisms underlying complex diseases. However, testing for gene-environment interaction remains a challenge since a.) statistical power is often limited and b.) modeling of environmental effects is nontrivial and such model misspecifications can lead to false positive interaction findings. To address the lack of statistical power, recent methods aim to identify interactions on an aggregated level using, for example, polygenic risk scores. While this strategy can increase the power to detect interactions, identifying contributing genes and pathways is difficult based on these relatively global results. Here, we propose RITSS (Robust Interaction Testing using Sample Splitting), a gene-environment interaction testing framework for quantitative traits that is based on sample splitting and robust test statistics. RITSS can incorporate sets of genetic variants and/or multiple environmental factors. Based on the user’s choice of statistical/machine learning approaches, a screening step selects and combines potential interactions into scores with improved interpretability. In the testing step, the application of robust statistics minimizes the susceptibility to main effect misspecifications. Using extensive simulation studies, we demonstrate that RITSS controls the type 1 error rate in a wide range of scenarios, and we show how the screening strategy influences statistical power. In an application to lung function phenotypes and human height in the UK Biobank, RITSS identified highly significant interactions based on subcomponents of genetic risk scores. While the contributing single variant interaction signals are weak, our results indicate interaction patterns that result in strong aggregated effects, providing potential insights into underlying gene-environment interaction mechanisms.

## Author summary

The understanding of gene-environment interactions provides potential insights into the pathways and mechanisms underlying complex diseases, but they are hard to detect since effect sizes are expected to be small. To facilitate the detection of such interactions in quantitative traits, we propose a robust and flexible approach called RITSS that can incorporate sets of genetic variants and multiple environmental factors. RITSS can utilize any suitable machine/statistical learning approach to screen for interactions and rigorously tests these aggregated signals using sample splitting and robust test statistics. We demonstrate the validity and power of our approach in extensive simulation studies. Furthermore, in an application to lung function and height data in the UK Biobank, RITSS discovers highly significant interactions based on subcomponents of genetic risk scores.

## Introduction

Genome-wide association studies (GWAS) have identified thousands of genetic variants associated with complex diseases and traits [1]. However, the effect of a genetic variant on a complex phenotype or disease can be modified by environmental exposures [2,3]. The knowledge about interactions between environmental exposures (e.g., tobacco smoke or occupational exposure) and genetic variants can provide insights into the underlying pathways and biological disease mechanisms [4].

Many of the methodological approaches for gene-environment interaction testing have focused on scenarios in which a single genetic variant and a single environmental variable are tested for potential interaction. Methods have been developed for case-control data, case-only data, and quantitative trait studies, summarized in detail by Gauderman et al. [5].

Since most gene-environment interaction effect sizes are expected to be small and statistical power is consequently limited [6], power-increasing approaches were proposed. This includes so-called screening statistics that prioritize genetic variants to reduce the multiple testing burden [6–14]. Also, researchers proposed to aggregate genetic information in a genomic region in set-based tests [15–23]. Recent approaches utilize mixed models, reaction norm models, or incorporate multiple environmental variables to derive a combination of environmental factors that potentially modifies the genetic effect [24–28].

Current data analyses observed that genetic risk scores, (weighted) sums of genetic risk variants, interact with smoking exposure in Coronary heart disease [29] and lung function [30,31]. While detecting significant interactions on this aggregated level is encouraging, identifying individual genes and pathways underlying these effects is challenging based on such results.

Besides the power limitation, another caveat of gene-environment interaction testing is that the misspecification of the marginal main effect models can lead to false positive findings in interaction testing [32,33]. This is especially problematic for continuous environmental factors where the implicit linearity assumption that is commonly used in the model might not be correct. One example is pack-years of smoking, where it is not straightforward to assume that every additional pack-year has the same, constant effect on the outcome.

In this communication, we propose RITSS, a robust and flexible framework for gene-environment interaction testing in quantitative traits. The general idea is to derive an interaction score comprised of the (weighted) sum of individual genetic variant/environmental factor product pairs, such that the combination of these signals increases the power while maintaining biological interpretability. Our approach utilizes a sample splitting strategy and test statistics that are robust against misspecifications of the main effects. The general form of RITSS allows the incorporation of user-specified screening/learning approaches in constructing candidate interaction scores while providing valid statistical inference without restrictive assumptions. In extensive simulation studies, we demonstrate the robustness and power of RITSS in various scenarios. We also applied RITSS to lung function phenotypes and human height in the UK Biobank, incorporating sex information and smoking exposure.

## Description of the method

### Ethics statement

This research was conducted by using the UK Biobank resource under application number 20915. All participants provided written informed consent, and study protocols were approved by the North West Multi-centre Research Ethics Committee and ethical procedures were controlled by the UK Biobank Ethics Advisory Committee.

### RITSS

In this section, we describe the RITSS framework and the corresponding algorithm.

### Set up

For sample *i*, we denote the quantitative trait by *Y*_*i*_, the *m*-dimensional genotype information by *X*_*i*_, and the *d*-dimensional environmental information by *E*_*i*_. Also, we define an additional *p*-dimensional covariate vector by *Z*_*i*_ that includes, for example, age, study indicators, and genetic ancestry principal components [34]. The genotype information *X*_*i*_ represents a pre-selected set of *m* genetic variants. We assume the following model

$$Y_{i}=\mu (E_{i},Z_{i})+\sum_{j=1}^{m}\pi_{j}(E_{i},Z_{i})X_{ij}+\varepsilon_{i}$$

where ***E***[*ε*_*i*_|*X*_*i*_, *Z*_*i*_, *E*_*i*_] = 0. The unknown function *μ* describes the main effect of the environmental factors *E*_*i*_ and other covariates *Z*_*i*_. The genetic contribution of variant *j* is modeled by *π*_*j*_(*E*_*i*_, *Z*_*i*_)*X*_*ij*_, where *π*_*j*_ is an unknown function. The null hypothesis of no gene-environment interaction is described by $E[Y_{i}|E_{i},Z_{i},X_{i}]=\mu (E_{i},Z_{i})+\sum_{j=1}^{m}\pi_{0j}(Z_{i})X_{ij}$, where *π*_0*j*_ is an unknown function depending on *Z*_*i*_ only. Therefore, we implicitly assume the absence of gene-gene-interactions but, in general, allow for interactions between the genetic variants and the covariates *Z*_*i*_ under the null hypothesis.

The rationale of RITSS is to incorporate sets of genetic variants and/or multiple environmental factors to increase power and detect interactions based on aggregated signals. This step is motivated by the observation that single variant approaches are often underpowered. There are two critical aspects that RITSS aims to address: 1.) testing of aggregated interaction signals can increase power, but the results are more difficult to interpret since individual factor contributions are unobserved, and 2.) recent results demonstrated that the misspecification of main effects in interaction analyses can lead to false positive interaction findings [32,33].

### Using sample splitting to screen for interaction signals

Given the input set of genetic and environmental factors, RITSS derives interaction scores of the form *U*_*i*_ = ∑_*j*_∑_*l*_*π*_*jl*_*X*_*ij*_*E*_*il*_ that combine signals adaptively while maintaining biological interpretability by keeping the number of involved factors of moderate size. RITSS uses sample splitting since screening and testing, in general, cannot be performed using the same data.

### Testing of interaction scores using a robust approach

Standard interaction tests are based on a product between the candidate interaction score and the phenotype adjusted for main effects ${\hat{Y}}_{i}^{resid}=Y_{i}-\hat{\mu }(E_{i},Z_{i})-\sum_{j=1}^{m}{\hat{\pi }}_{0j}{(Z_{i})X}_{ij}$. Zhang et al. showed that, for a quantitative trait (identical link function), main effects misspecifications can lead to invalid interaction tests, especially if genetic variants and environmental factors are not independent [33]. To avoid main effect misspecifications, they proposed to apply generalized additive models (GAMs) to model the main effects flexibly. RITSS follows this idea and incorporates rich models for the main effects, including GAM-based solutions. However, while Zhang et al. considered scenarios of single variant and single environmental factors, the dimensions of *X*_*i*_, *E*_*i*_, and *Z*_*i*_, that is *m*, *d*, and *p*, in our setting are of substantial size. The potential high dimensionality of the models leads to slower convergence rates so that standard results might not apply here. Furthermore, considering RITSS as a general framework, we aim to derive an algorithm that can incorporate any other suitable future approach. Therefore, we utilize a concept based on recent theoretical results in causal inference [35]. Specifically, instead of considering the standard test statistic $\sum_{i}U_{i}{\hat{Y}}_{i}^{resid}$, RITSS is based on $\sum_{i}{\hat{U}\prime }_{i}{\hat{Y}}_{i}^{resid}$, where ${\hat{U}\prime }_{i}$ is an estimate of a transformation of *U*_*i*_ that is orthogonal to (i.e., has covariance zero with) the environmental and genetic main effects. This orthogonality improves the robustness against main effect model misspecifications since it reduces the impact of residual main effects in ${\hat{Y}}_{i}^{resid}$ on the product test statistic. Furthermore, we extend the sample splitting and estimate the main effect models as well as the transformation in non-overlapping subsamples of the data that also do not overlap with the subsamples used for the screening step and the subsamples used for evaluating the test statistic. This enables to apply any suitable approach for the screening step and the estimation of the main effect models/transformations, without the need to characterize the asymptotic behavior. It reduces the necessary assumptions to suitable convergence rates [36].

### Algorithm

**Fig 1:**
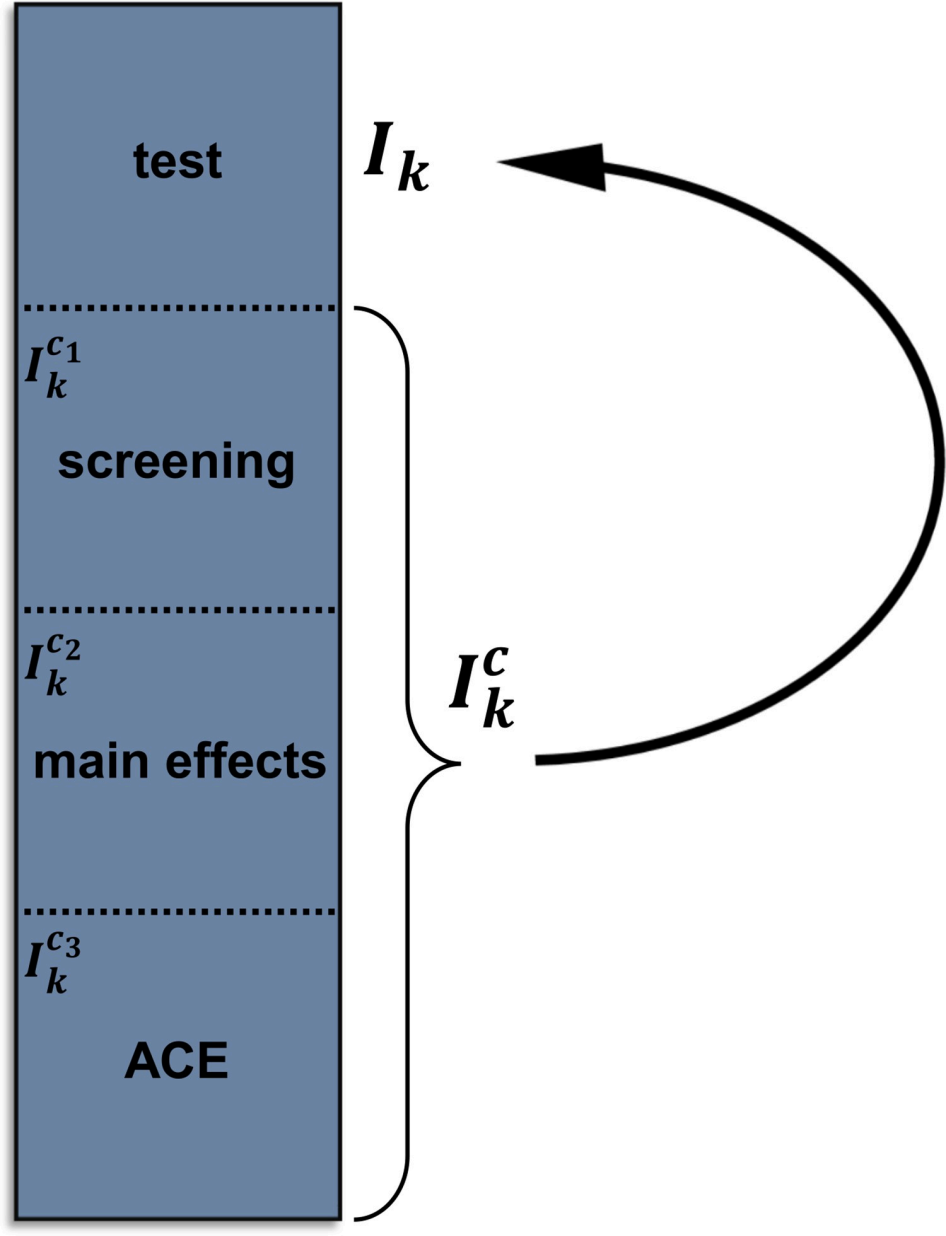
Visualization of the RITSS algorithm.

We here describe the algorithm underlying RITSS. The algorithm is also visualized in Fig 1.

**Input:** data *X*_*i*_, *E*_*i*_, *Z*_*i*_, *Y*_*i*_, screening function *Screen*(*S*) including the parameter *S* (number of candidate scores), parameter *K* for the number of splits, splitting fractions *c* = (*c*_1_, *c*_2_, *c*_3_).

**Algorithm:**

- Split the data (*Y*_*i*_, *X*_*i*_, *E*_*i*_, *Z*_*i*_) randomly into *K* non-overlapping subsamples *I*_*k*_, *k* = 1,…,*K*, of approximately equal size.
- For each subsample *I*_*k*_:
  - Denote all samples not in *I*_*k*_ by $I_{k}^{c}$ and split $I_{k}^{c}$ randomly into 3 non-overlapping parts $I_{k}^{c1},I_{k}^{c2},$ and $I_{k}^{c3}$, according to the splitting fractions *c*.
  - Perform interaction signal screening based on $I_{k}^{c1}$ using the screening function *Screen*(*S*) to obtain *S* candidate interaction scores $U_{is}=\sum_{j_{s}}\sum_{l_{s}}\pi_{j_{s}l_{s}}^{s}X_{ij_{s}}E_{il_{s}}$, *s* = 1,…,*S*.
  - In $I_{k}^{c2}$, fit the interaction scores *U*_*is*_, *s* = 1,…,*S*, as predictors in a linear model while incorporating flexible models for the main effects $\hat{\mu }(E_{i},Z_{i})$ and $\sum_{j=1}^{m}{\hat{\pi }}_{0j}{(Z_{i})X}_{ij}$. Select all or a subset of scores based on user-specified criteria and combine these scores into a single interaction score *U*_*i*_.
  - Using the alternating conditional expectation (ACE) algorithm [37], estimate/derive the orthogonalized transformation $U_{i}^{\prime }$ of *U*_*i*_ in $I_{k}^{c3}$ (S1 Appendix), denoted by ${\hat{U}\prime }_{i}$.
  - Based on the predictions of the trained models, compute $T_{k}=\sum_{i\epsilon I_{k}}{\hat{U}\prime }_{i}{\hat{Y}}_{i}^{resid}$ and ${\hat{\sigma }}_{k}^{2}=\sum_{i\epsilon I_{k}}{\left[{\hat{U}\prime }_{i}{\hat{Y}}_{i}^{resid}\right]}^{2}$ in *I*_*k*_.
- Compute the overall statistic $T=\sum_{k}T_{k},\;{\hat{\sigma }}_{T}^{2}=\sum_{k}{\hat{\sigma }}_{k}^{2}$, and the z-score $z=\frac{T}{\sqrt{{\hat{\sigma }}_{T}^{2}}}$.

## Properties and discussion

Under the null hypothesis of no gene-environment interaction, the overall z-score *z* is asymptotically normal, that is *z*→*N*(0,1) [35,38] and an overall RITSS interaction p-value can be computed. This theoretical result applies if, besides standard regularity conditions, the main effects $\hat{\mu }(E_{i},Z_{i})$+$\sum_{j=1}^{m}{\hat{\pi }}_{0j}{(Z_{i})X}_{ij}$ and the transformations ${\hat{U}\prime }_{i}$ are estimated with sufficiently fast convergence rates (as a function of sample size). These assumptions are closely related to the corresponding dimensions of genetic information, environmental factors, and covariates (*m*, *d*, and *p*, respectively), since the increasing dimensions increase the complexity of estimating these models sufficiently precise. This is where the advantage of the robust approach is apparent over, for example, solely application of generalized additive models (GAMs). The robust approach reduces the required assumptions to the statement that the specific products of convergence rates (main effects and transformation) are fast enough instead of requiring fast convergence rates for the main effects directly. As a remark, if we assume the absence of interactions between genetic variants and the covariates *Z*_*i*_ and an additive genetic model, that is *π*_0*j*_(*Z*_*i*_) = *π*_0*j*_, the genetic main effect model becomes less complex, and faster convergence rates can be achieved. Nevertheless, since general models become complex with growing dimensions rapidly, we consider sets of *m* genetic variants with *m* in the magnitude of 10^2^−10^3^ and *d*≤10 and *p*≤10 as suitable input dimensions for RITSS.

We emphasize that it is therefore only required to estimate the transformation $U_{i}^{\prime }$ of a given interaction score *U*_*i*_ accurately, but the interaction score *U*_*i*_ itself can be constructed using any suitable method in the screening step, including machine learning or other advanced statistical learning procedures. Under the null hypothesis, the test is ensured to be valid.

On the other hand, the power of RITSS depends on the ability of the screening strategy to detect signals and capture them in the interaction score *U*_*i*_. Therefore, although the screening step can be implemented flexibly, it is crucial for statistical power. We discuss and investigate two different screening strategies in the next section. It is important to note that the *K* different interaction scores *U*_*i*_, in general, will include different interaction factors since the corresponding screening was performed in different parts of the data. Investigating the overlap between the interaction scores provides further fine mapping of the results.

### Specific implementation

The screening strategy influences the statistical power of RITSS. There is no uniformly optimal strategy since the optimal screening depends on the interaction signal architecture. Specific implementations of RITSS differ in the realization of steps b.) and c.); steps a.), d.), and e.) are fixed throughout different implementations. Here, we describe two different implementations; we will refer to them as RITSS1 and RITSS2. These two implementations will be investigated in the simulation studies in the next section.

### Screening strategy 1: interaction between single environmental factor and subcomponent of genetic risk score

The first screening strategy aims to identify subcomponents of genetic risk scores that interact with a single environmental factor *E*_*it*_, where *t* is the index of the selected factor. In step b.), we first fit the model $Y_{i}=\mu_{0}+{E_{i}^{T}\mu }_{1}+Z_{i}^{T}\mu_{2}+X_{i}^{T}\pi_{0}+\varepsilon_{i}$ in $I_{k}^{c1}$ to obtain estimated genetic main effects ${\hat{\pi }}_{0j}$. Then, we perform approximate best subset selection [39] to identify sets *J*_*s*_, *s* = 1,2, that capture interaction scores of the form $U_{is}=\sum_{j\in J_{s}}{\hat{\pi }}_{0j}X_{ij}E_{it}$, describing subcomponents of genetic risk scores multiplied with the environmental factor *E*_*it*_. The best subset selection is performed using a 2-sample split of $I_{k}^{c1}$ and a step size of 5 between 10 and the maximum number of variants available after additional filtering. *J*_1_ contains all variants that intersect between the best subsets, *J*_2_ contains all variants that were included exactly once. More details are described in S1 Appendix.

In step c.), we construct the overall interaction score *U*_*i*_ = *U*_*i*1_+*c*_2_*U*_*i*2_, where *c*_2_ is set to 1, if the corresponding regression coefficient p-value is below 0.05, otherwise *c*_2_ = 0. We denote the set of genetic variants in the interaction score *U*_*i*_ that is tested in *I*_*k*_ by *m*(*I*_*k*_).

### Screening strategy 2: aggregated single environmental factor interactions based on single variant testing

In step b.), we fit the models $Y_{i}=\mu_{0}+{E_{i}^{T}\mu }_{1}+Z_{i}^{T}\mu_{2}+X_{ij}\pi_{0j}+E_{it}X_{ij}\pi_{jt}+\varepsilon_{i}$ and select variants together with the estimated ${\hat{\pi }}_{jt}$ into the at most two interaction scores based on False Discovery Rate (FDR) q-values to correct for multiple testing. Step c.) is implemented as in screening strategy 1. We refer to S1 Appendix for more details. As for the screening strategy 1, we denote the set of genetic variants in the interaction score *U*_*i*_ that is tested in *I*_*k*_ by *m*(*I*_*k*_).

## Verification and comparison

In this section, we describe the simulation studies and the application of RITSS to lung function and human height data in the UK Biobank.

### Simulation studies

We performed extensive type 1 error and power simulations in which we investigated the performance of RITSS and compared our approach with alternative approaches. We emphasize that the power of RITSS depends on the combination of the underlying interaction signal architecture and the implemented screening strategy. Various screening strategies can be used, and different screening approaches are suitable for different signal architectures. In all simulation studies, we used the two specific implementations RITSS1 and RITSS2 described above. Both strategies considered the single environmental factor *E*_*it*_ with *t* = 1 for interaction scores. Moreover, we applied RITSS1 and RITSS2 with *K* = 3, 4 and the splitting fractions *c* = (0.5, 0.25, 0.25) as well as *c* = (1/3, 1/3, 1/3), to investigate the impact of these choices. Also, the implemented main effect models assume no gene-covariate interactions and an additive genetic model under the null hypothesis.

### Alternative approaches

We included two alternative gene-environment interaction testing approaches in our simulation studies. The rationale is to demonstrate the differences and highlight the advantages of RITSS compared to existing and established methods.

1. We included GESAT, a variance component based test [17]. For GESAT, we considered *E*_*it*_ with *t* = 1 as the potential interaction factor, and *Z*_*i*_ as well as *E*_*ij*_, *j* = 2,…,*d* as covariates. We note that GESAT tests all *m* genetic variants jointly for interaction with *E*_*i*1_; GESAT does not perform sample splitting and does not aim to identify the subset of variants that is involved in the interaction.
2. We implemented a single variant-based approach in which the environmental main effects are modelled using GAMs. Specifically, for each genetic variant, a GAM is fitted with the genetic variant and the interaction term as standard covariates, together with the flexible spline-based part for the environmental main effect (see S1 Appendix for more details). The *m* interaction p-values are summarized by the minimum p-value after Bonferroni correction for *m* tests. This approach will be denoted by GAMsv.

Furthermore, besides GESAT and GAMsv, we also included a modified version of RITSS1 and RITSS2 in which the test statistic utilizes the interaction scores *U*_*i*_ directly, instead of the robust version based on the transformed $U_{i}^{\prime }$. The rationale is to demonstrate the necessity of this step in the high-dimensional setting; this approach will be denoted by D1 and D2, respectively.

### Simulation settings: type 1 error

In all scenarios, we simulated a sample size of *n* = 30,000, *m* = 100 SNPs, *d* = 5 environmental factors *E*_*i*_, and *p* = 2 covariates *Z*_*i*_. The phenotype *Y*_*i*_ was simulated based on $Y_{i}=\mu (E_{i},Z_{i})+X_{i}^{T}\pi_{0}+\varepsilon_{i}(E_{i},Z_{i})$ with ***E***[*ε*_*i*_(*E*_*i*_, *Z*_*i*_)|*X*_*i*_, *Z*_*i*_, *E*_*i*_] = 0.

Under the null hypothesis of no gene-environment interaction, the overall RITSS z-score is asymptotical normal with *z*→*N*(0,1). The type 1 error simulations aim to demonstrate the accuracy of this asymptotic approximation in realistic scenarios.

As described in the methods section, the screening strategies of RITSS1 and RITSS2 assume linear environmental main effects. We included scenarios in which the true environmental main effect is non-linear and gene-environment correlation is present. In these scenarios, the screening strategies, therefore, construct interaction scores that are reflecting false positive interaction findings [32,33]. Besides, we included scenarios with non-normal and/or heteroscedastic random errors in the phenotype model. In total, we simulated five different scenarios: (1): no misspecifications, normal homoscedastic errors, (2): mis specified environmental main effects in the screening strategies, (3): mis specified environmental main effects in the screening strategies in combination with non-normal heteroscedastic errors, (4): scenario 2 including gene-environment correlations, and (5): scenario 3 including gene-environment correlations.

In addition, we investigated if the type 1 error rate is inflated in the case where the genetic variants for the analysis were selected based on GWAS results in the same dataset, i.e., based on their marginal association p-values (scenario notated as SELECT:yes/no). This is of particular interest since many reported genetic associations were identified based on the large-scale datasets that are suitable for gene-environment interaction testing. In total, this resulted in 10 different type 1 error scenarios (scenarios 1–5, SELECT:yes/no).

The specific details of the implementations of these scenarios are described in S1 Appendix. The type 1 error simulations are based on 10,000 replicates.

### Simulation settings: power

In addition to the type I error simulations, we also included a set of power simulations. In the power simulations, we considered scenario 1 with SELECT:no from the type 1 error simulations but incorporated a gene-environment interaction component. The interaction contribution is controlled by the mean *μ*_*XE*_ and standard deviation *σ*_*XE*_ of interaction effects, as well as the density *p*_*XE*_ of these interaction effects. The interaction model incorporates the first component *E*_*i*1_ only. The detailed implementation is described in S1 Appendix. The power simulations are based on 1,000 replicates.

### Results: the choice of *K* and the splitting fractions *c*

RITSS can be applied using different choices for *K* and the splitting fractions *c*. While the asymptotic theory, that is the asymptotic standard normality of the RITSS z-score under the null hypothesis, applies for any fixed choice, the finite sample performance in practice as well as the power can be influenced by these choices. Choosing these parameters faces specific tradeoffs. For a fixed *K*, splitting fractions *c* that allocate more sample size to the screening strategy are expected to have higher power, while the decreased sample size for the main effect and transformation estimation might lead to less accurate model fits and therefore inflated type 1 error rates. Since every sample contributes exactly once to the test statistic for each fixed *K*, increasing *K* (moderately) is expected to increase power since a bigger fraction of the overall sample is used for screening. At the same time, a larger *K* can lead to increased computational burden, depending on how the screening and main effect model estimation tasks scale with sample size. We considered the four combinations of *K* = 3, 4 and the splitting fractions *c* = (0.5, 0.25, 0.25) and *c* = (1/3, 1/3, 1/3). In Table B in S1 Appendix, we report initial type 1 error results for scenarios 1–5 with SELECT:no for all four combinations based on 1,000 replicates. Table C in S1 Appendix summarizes power results for selected scenarios based on 1,000 replicates.

The results in Table B in S1 Appendix show that, although all four combinations provide approximately uniformly distributed p-values under these null hypothesis scenarios, the splitting fraction *c* = (1/3, 1/3, 1/3) leads to the most stable results; this is in line with the intuition described above. Furthermore, D1 and D2 do not control the type 1 error rates, demonstrating that the robust test statistics are needed to provide valid results. The power results in Table C in S1 Appendix demonstrate that *K* = 4 has slightly more power than *K* = 3, as intuitively described above, but the overall power differences between the four combinations are small. For the full type 1 error and power simulations, we considered the combination of *K* = 4 and *c* = (1/3, 1/3, 1/3).

### Results: type 1 error

**Fig 2:**
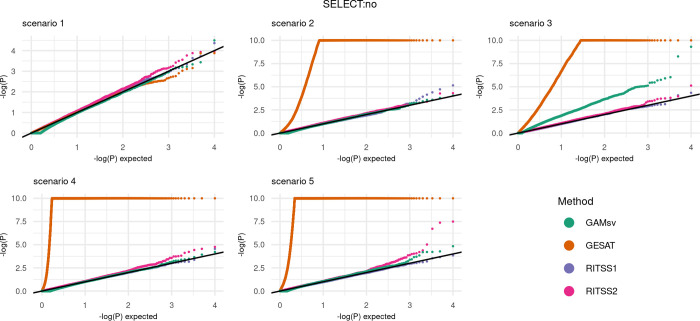
Type 1 error: Quantile-quantile-plots for RITSS1, RITSS2, GESAT, and GAMsv for scenarios 1–5 with SELECT:no. All results based on 10,000 replicates. P-values with *p*<10^−10^ were set to *p* = 10^−10^. SELECT:no refers to the scenario where all simulated genetic variants are included in the analysis.

**Fig 3:**
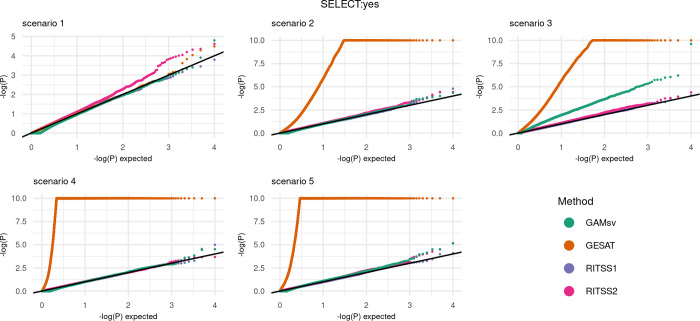
Type 1 error: Quantile-quantile-plots for RITSS1, RITSS2, GESAT, and GAMsv for scenarios 1–5 with SELECT:yes. All results based on 10,000 replicates. P-values with *p*<10^−10^ were set to *p* = 10^−10^. SELECT:yes refers to the scenario where the simulated genetic variants are selected into the analysis based on marginal association p-values.

We report the quantile-quantile-plots (qq-plots) for scenarios 1–5 with SELECT:no in Fig 2, as well as the qq-plots for scenarios 1–5 with SELECT:yes in Fig 3.

Overall, the simulations demonstrate that the results for SELECT:no and SELECT:yes are similar. RITSS1 and RITSS2 provide controlled type 1 error rates across all five scenarios. GESAT demonstrates valid results for scenario 1 but is highly inflated in the scenarios 2–5. We note that we cut off the GESAT p-values at 10^−10^ in the plots for better visualization. The inflated results are expected since GESAT does not account for heteroscedastic errors (scenarios 3 and 5) and the linear main effect model implies a mis specification in scenarios 2–5. GAMsv, which models the environmental main effects similar to RITSS, shows valid type 1 error rates for scenarios 1, 2, 4, and 5. In scenario 3, the heteroscedastic errors lead to inflation. In summary, our type 1 error simulations demonstrate the necessity of flexible main effect models to ensure robustness and that RITSS provides valid type 1 error rates in all investigated scenarios.

### Results: power

**Fig 4:**
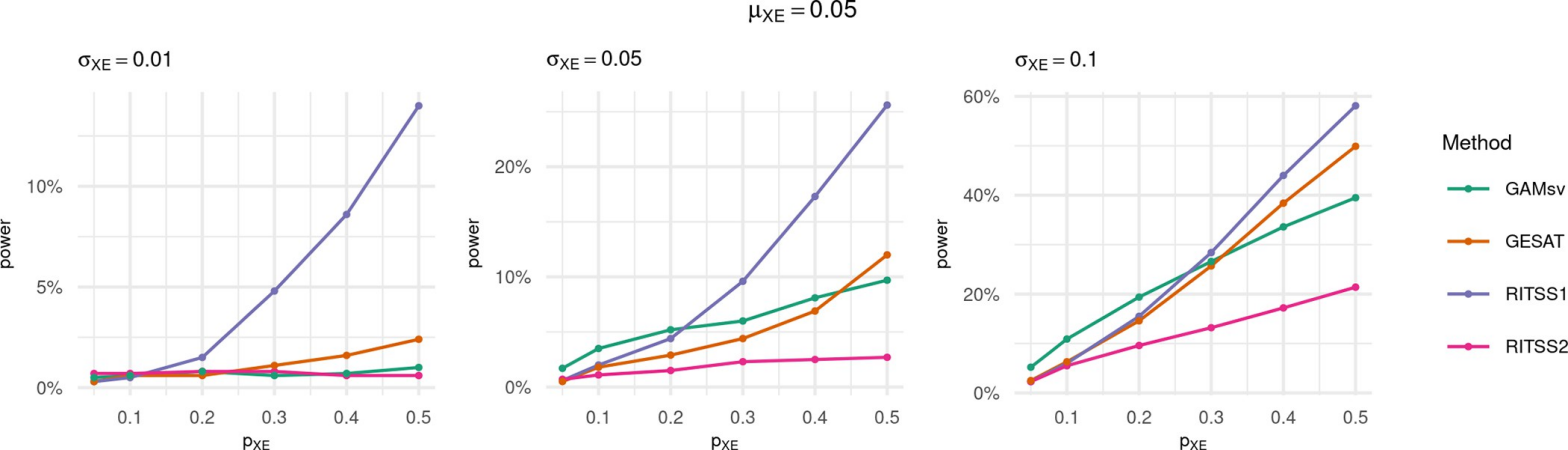
Power curves for RITSS1, RITSS2, GESAT, and GAMsv over increasing signal density *p*_*XE*_. Significance level *α* = 0.005, *μ*_*XE*_ = 0.05, and results based on 1,000 replicates. Power was simulated for *p*_*XE*_ = 0.05, 0.1, 0.2, 0.3, 0.4, 0.5.

**Fig 5:**
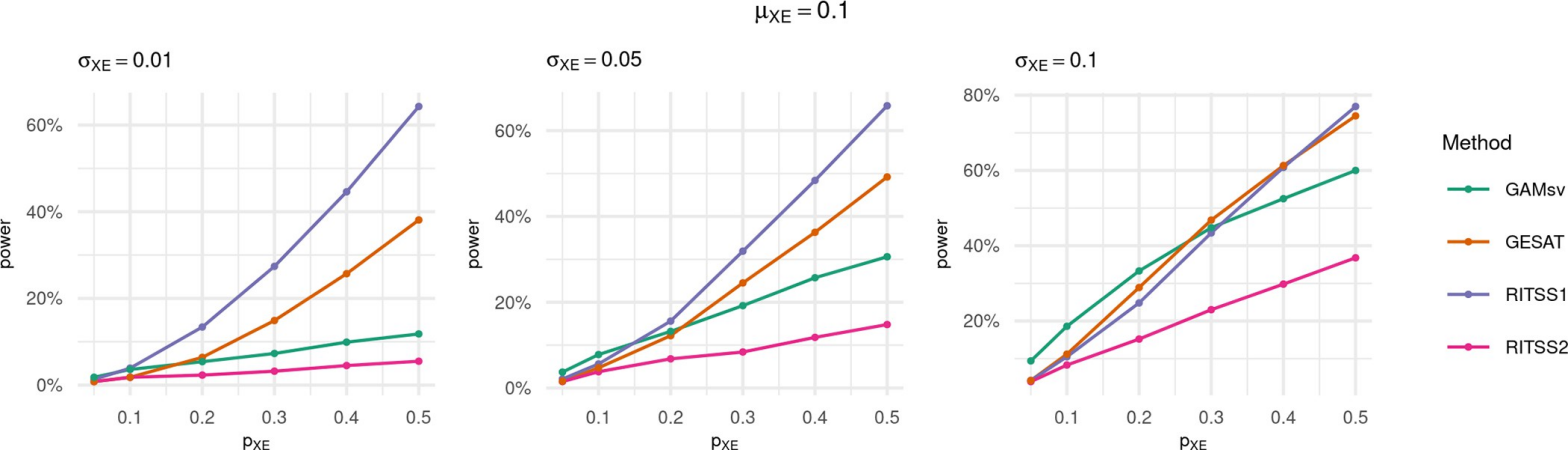
Power curves for RITSS1, RITSS2, GESAT, and GAMsv over increasing signal density *p*_*XE*_. Significance level *α* = 0.005, *μ*_*XE*_ = 0.1, and results based on 1,000 replicates. Power was simulated for *p*_*XE*_ = 0.05, 0.1, 0.2, 0.3, 0.4, 0.5.

The results of the power simulations are reported in Figs 4 and 5, where the power is displayed as a curve over increasing signal density *p*_*XE*_, for different effect size distributions. The significance level was set to *α* = 0.005.

We consider the results for *μ*_*XE*_ = 0.05 in Fig 4 first. We observe that RITSS1 is more powerful than RITSS2 with increasing signal density. This is expected since the simulated signal architecture represents interactions with subcomponents of risk scores, and the screening strategy of RITSS1 aims to detect exactly these signals.

RITSS1 outperforms GESAT and GAMsv in most scenarios, especially for *σ*_*XE*_ = 0.01 and increasing *p*_*XE*_. The power difference is smaller for *σ*_*XE*_ = 0.1. With larger effect sizes and sparse signals, GAMsv shows advantages since the single variant tests are powerful enough to circumvent the multiple testing burden in this case.

The results in Fig 5 for *μ*_*XE*_ = 0.1 are similar, but the power advantages of RITSS1 are smaller or vanish (*σ*_*XE*_ = 0.1). Overall, the power simulations demonstrate the interplay between the screening strategy implemented in RITSS and the resulting power. Especially for dense signals with small effect sizes, RITSS1 shows substantial power advantages. We hypothesize that these signal architectures approximate the signals observed in recent interaction studies based on polygenic risk scores.

### Applications

We applied RITSS to UK Biobank data to analyze gene-environment interactions in lung function (measured by forced expiratory volume in 1 second (FEV_1_), forced vital capacity (FVC), and the ratio FEV_1_/FVC) and height. Here, for our analyses, we restricted the input to previously reported GWAS associations for the respective trait. This step reduces the dimensionality of *X*_*i*_, denoted by *m*, to a range between 383 and 3368. We note that this choice can be too restrictive since interaction effects do not need to be accompanied by main effects. Details about the study population as well as the extraction of genetic, environmental, and phenotypic data are described in S1 Appendix.

### Analysis setup

Table 1 contains the configurations of *Y*_*i*_, *X*_*i*_, *E*_*i*_, and *Z*_*i*_ for the four different phenotype analyses. Age and pack-years of smoking (P-Y-S) were mean-centered before computing the squared variable. Sex was coded as male = 1 and female = 0 in this analysis. For the lung function traits, we also included height in *E*_*i*_ as a potentially interacting variable. We note that, given the coding of sex in this analysis, height and sex are (strongly) positively correlated. The lung function measurements were analyzed on the original scale and not transformed, as robustness of the approach against non-normal errors was demonstrated in the type 1 error simulations. After quality control, we split the resulting 254,033 samples (European ancestry) in two parts: 180,000 randomly selected samples for the main analysis using RITSS and 74,033 samples that serve as an independent dataset to validate the analysis results of RITSS. We applied RITSS using the same two different implementations described in the simulation studies (RITSS1 and RITSS2). For both RITSS1 and RITSS2, we chose *K* = 4 and $c=(\frac{1}{3},\frac{1}{3},\frac{1}{3})$. Each of the factors in *E*_*i*_ was tested for interaction separately, resulting in a total of 32 tests. In S1 Fig, we plotted the estimated densities of the standardized residuals for each phenotype after adjusting for *X*_*i*_, *E*_*i*_, and *Z*_*i*_ using standard linear regression. The density plots are based on the 180,000 samples in the main analysis.

**Table 1:** Analysis configurations and number of genetic variants incorporated.

*Y*_*i*_	*X*_*i*_	*E*_*i*_	*Z*_*i*_
FEV_1_	383 SNPs	sex, height, P-Y-S, E-S, P-Y-S^2^	age, age^2^, PCs 1–10, genotyping-array
FVC	638 SNPs	sex, height, P-Y-S, E-S, P-Y-S^2^	age, age^2^, PCs 1–10, genotyping-array
FEV_1_/FVC	885 SNPs	sex, height, P-Y-S, E-S, P-Y-S^2^	age, age^2^, PCs 1–10, genotyping-array
height	3368 SNPs	sex	age, age^2^, PCs 1–10, genotyping-array

P-Y-S: pack-years of smoking, E-S: ever-smoking, PCs: genetic ancestry principal components. FEV_1_: forced expiratory volume in 1 second, FVC: forced vital capacity (FVC). Pack-years of smoking and age were centered before computing the respective squared variable.

## Results

Table 2 contains the results of our UK Biobank main analysis using 180,000 samples based on RITSS1 and RITSS2. For each of the four traits, we observed a highly significant interaction between sex and subcomponents of the genetic risk score using RITSS1. For the lung function traits, we also observed similar interaction findings with height. We investigated this in more detail in the validation analysis below.

**Table 2:** Results of the interaction testing using the two approaches RITSS1 and RITSS2 in the UK Biobank. The environmental factor tested for interaction is denoted by *E*_*it*_. |*m*| is the number of total SNPs in the analysis, |*m*_4_| and |*m*_3_| are the number of SNPs that are shared by all four and exactly three interaction scores, respectively. P-Y-S: pack-years of smoking, E-S: ever-smoking.

*Y*_*i*_	*E*_*it*_	m	RITSS1 p-value	|*m*_4_|	|*m*_3_|	RITSS2 p-value	|*m*_4_|	|*m*_3_|
FEV_1_	sex	383	2.86E-25	44	54	7.68E-01	0	0
FEV_1_	height	383	1.75E-13	56	52	4.81E-01	0	0
FEV_1_	P-Y-S	383	8.81E-02	1	3	6.37E-01	0	0
FEV_1_	E-S	383	6.08E-01	0	12	4.16E-01	0	0
FEV_1_	P-Y-S^2^	383	5.41E-02	0	2	8.77E-01	0	0
FVC	sex	638	1.27E-22	46	94	1.76E-01	0	0
FVC	height	638	1.07E-21	54	101	5.10E-01	0	0
FVC	P-Y-S	638	6.08E-01	1	1	4.51E-01	0	0
FVC	E-S	638	1.19E-01	1	4	4.02E-01	0	0
FVC	P-Y-S^2^	638	6.77E-01	0	0	9.77E-01	0	0
FEV_1_/FVC	sex	885	7.33E-22	83	106	3.91E-03	0	0
FEV_1_/FVC	height	885	8.41E-16	74	119	9.13E-01	0	0
FEV_1_/FVC	P-Y-S	885	2.01E-04	78	100	2.61E-01	0	0
FEV_1_/FVC	E-S	885	6.35E-03	74	106	5.56E-04	0	0
FEV_1_/FVC	P-Y-S^2^	885	1.07E-01	0	1	2.35E-01	0	3
height	sex	3368	1.51E-20	210	341	2.78E-01	0	0

Furthermore, in line with previous results in the literature, we observed a significant interaction with pack-years of smoking in FEV_1_/FVC [30,31] that maintains significant after Bonferroni correction for *n* = 32 tests at a significance level of *α* = 0.05.

Based on the *K* = 4 different interaction scores, we investigated the overlap between the genetic variants included in the sets *m*(*I*_*k*_), *k* = 1,…,4. We denote all genetic variants that were in all four scores by *m*_4_ and all genetic variants included in exactly three scores by *m*_3_. For the validation analysis, we considered all *Y*_*i*_/*E*_*it*_ pairs that had a Bonferroni corrected significant interaction p-value at a significance level of *α* = 0.05 and non-empty *m*_4_ or *m*_3_ sets; this included RITSS1 results only.

The validation analysis was based on the remaining and independent 74,033 samples and a standard regression interaction test while fitting *X*_*i*_, *E*_*i*_, and *Z*_*i*_. We tested two interaction scores, the first based on the variants in the corresponding *m*_4_ set and the second based on variants in *m*_3_. Here, the genetic main effects for the genetic risk scores were estimated based on the 180,000 samples in the main analysis. The regression interaction p-values were evaluated based on model-based standard errors as well as standard errors obtained from a robust sandwich estimator.

The results of our validation analysis are described in Table 3. The results show that all findings replicate. We also note that the directions of effects were consistent between the main analysis and validation analysis.

**Table 3:** Results of the validation analysis in the 74,033 remaining samples in the UK Biobank. The two sets *m*_4_ and *m*_3_ were identified in the corresponding main analysis (Table 2) and correspond to the results of the RITSS1 analysis. The interactions between the scores (corresponding to *m*_4_ and *m*_3_ respectively) and the environmental factor *E*_*it*_ were tested using a linear regression, adjusting for all *X*_*i*_, *E*_*i*_ and *Z*_*i*_. The robust p-values are based on standard errors obtained by a robust sandwich variance estimator. P-Y-S: pack-years of smoking.

*Y*_*i*_	*E*_*it*_	p-value *m*_3_	robust p-value *m*_3_	|*m*_3_|	p-value *m*_4_	robust p-value *m*_4_	|*m*_4_|
FEV_1_	sex	9.11E-06	2.68E-05	54	3.41E-10	1.65E-09	44
FEV_1_	height	8.26E-07	2.30E-06	52	2.19E-09	1.81E-08	56
FVC	sex	1.48E-07	5.61E-07	94	5.29E-07	1.68E-06	46
FVC	height	1.96E-07	9.79E-07	101	5.04E-10	6.96E-09	54
FEV_1_/FVC	sex	7.23E-04	8.08E-04	106	4.88E-07	6.80E-07	83
FEV_1_/FVC	height	4.95E-05	6.63E-05	119	1.36E-06	1.82E-06	74
FEV_1_/FVC	P-Y-S	1.21E-04	2.91E-03	100	2.22E-07	6.31E-05	78
height	sex	1.46E-02	1.56E-02	341	2.07E-02	2.19E-02	210

To disentangle these findings in more detail, we examined the single variant interaction p-values for the genetic variants in the combined set *m*_4_/*m*_3_ with the respective environmental factor in the validation dataset. This analysis was based on a linear regression that fits *X*_*i*_, *E*_*i*_, and *Z*_*i*_, and the respective interaction term. Table 4 lists the number of genome-wide significant single variant interactions, i.e., *p*<5×10^−8^, as well as the number of significant single variant interactions using a Bonferroni correction adjusting for the number of variants in the corresponding combined set *m*_4_/*m*_3_ at a significance level of *α* = 0.05. Both numbers are either 0 or very small, for all trait/environmental factor combinations. This suggests that the interaction effects at the single variant level are weak. However, we made an interesting observation by investigating the empirical distribution of directions of effect across the single variant interaction tests. Table 4 provides these empirical distributions of directions of effect for a.) the unweighted interaction terms *X*_*ij*_×*E*_*it*_ and b.) the weighted terms ${\hat{\pi }}_{0j}*X_{ij}*E_{it}$, where ${\hat{\pi }}_{0j}$ is the estimated genetic effect based on the main analysis dataset. In addition, we compared the interaction p-value distributions according to the directions of effect of the weighted interaction terms. As we can see by the enrichment of positive effects, the weighting by the genetic main effect, as it is applied in the computation of the genetic risk score, “aligns” the interaction effect directions across the genetic variants. Furthermore, the p-values for weighted interaction terms with positive effect show substantially higher inflation. This indicates that RITSS1 identified genetic variants whose interaction signals are weak at the single variant level but when summed up in the genetic risk score, they lead to a significant interaction. Since we used a coding of sex of male = 1 and female = 0, this suggests that the genetic effects in males and females across these variants have the same direction, but the magnitudes of effect in males are slightly larger. Related, we note that the genetic variants in *m*_4_/*m*_3_ between the sex and height interactions in lung function highly overlap, which is in line with the observation that height and sex are strongly positively correlated. However, if we test the height interaction separately in males and females in the validation dataset, the signal vanished or was strongly diminished. Therefore, we hypothesize that the observed interactions are a result of sex-differential effects, but further analyses are required to disentangle the exact mechanisms. For example, investigating the impact of sitting or thoracic height, instead of standing height [40]. In this context, we note that, related to recent results regarding so-called participation biases and their impact on sex-related analyses [41], the genetic variants in *m*_4_/*m*_3_ across the three lung function phenotypes and height were not significantly associated with sex, but replication of the interaction signal in an independent cohort is preferable. Detailed information about the selected variants in the gene-by-sex interactions for lung function and height can be found in S2 Appendix.

**Table 4:** Results of the validation analysis in the 74,033 remaining samples in the UK Biobank II. The two sets *m*_4_ and *m*_3_ were identified in the corresponding main analysis based on the RITSS1 approach (Table 2). All variants in *m*_4_/*m*_3_ were tested for interaction with the respective environmental factor *E*_*it*_. In this context, gws. interaction refers to a single variant interaction p-value *p*<5×10^−8^. Similar, bfs. interaction corresponds to *p*<0.05/(|*m*_4_|+|*m*_3_|) (Bonferroni correction). The last columns report the empirical distribution of effect directions in these tests and the corresponding 5% quantile of p-values (weighted analysis). In the weighted analysis, the genotype/env.factor product term was multiplied by the estimated genetic main effect (obtained from the independent main dataset). P-Y-S: pack-years of smoking.

*Y*_*i*_	*E*_*it*_	|*m*_3_|+|*m*_4_|	No. gws interactions	No. bfs. interactions	Distribution effect directions unweighted -/+ (in %)	Distribution effect directions weighted -/+ (in %)	5% quantile interaction p-values in weighted -/+
FEV_1_	sex	98	0	1	53/47	23/77	1.636e-01/8.190e-03
FEV_1_	height	108	0	1	51/49	29/71	7.963e-02/7.341e-03
FVC	sex	140	0	0	46/54	26/74	3.493e-02/1.107e-02
FVC	height	155	0	0	43/57	31/69	4.220e-02/1.103e-02
FEV_1_/FVC	sex	189	0	0	51/49	37/63	1.730e-01/1.962e-02
FEV_1_/FVC	height	193	0	0	48/52	39/61	1.478e-01/2.565e-02
FEV_1_/FVC	P-Y-S	178	0	0	46/54	36/64	6.412e-02/1.051e-02
height	sex	551	0	0	49/51	45/55	5.655e-02/3.849e-02

We also applied GESAT in the validation analysis. For each of the four traits, we applied GESAT to test for interaction with sex (using the remaining *E*_*i*_ and *Z*_*i*_ as covariates) based on 1.) all genetic variants in the analysis and 2.) all genetic variants in the corresponding combined *m*_4_/*m*_3_ set. For all traits, these tests were at least nominal significant (*p*<0.05), and we obtained similar p-values in the two analyses 1.)/2.) (except for FVC): FEV_1_: *p* = 1.81×10^−11^/*p* = 8.92×10^−7^, FVC: *p* = 2.71×10^−14^/*p* = 2.29×10^−4^, FEV_1_/FVC: *p* = 2.61×10^−2^/*p* = 2.29×10^−2^, height: *p* = 1.08×10^−3^/*p* = 6.19×10^−3^. These results add further evidence that RITSS1 identified a fine mapped set of interacting genetic variants.

## Discussion

We propose RITSS, a robust and flexible interaction testing framework for quantitative traits. Our framework aims to identify aggregated interaction signals and tests these signals using sample splitting and robust test statistics. Since interactions at the single genetic variant level are hard to detect due to small effect sizes, we hypothesize that strategies that aggregate suitable signals across a limited number of factors/loci have higher statistical detection power while, at the same time, they still permit an interpretation and can reveal potential biological mechanisms.

In extensive simulations, we investigated the performance of RITSS based on two distinct implementations and demonstrated that our framework controls the type 1 error rates across different scenarios. In contrast, alternative approaches showed inflated type 1 error rates in these scenarios. Additionally, we demonstrated the interplay between the screening strategy and RITSS’s power based on selected power simulations. We applied our approach to UK Biobank data and observed potential interactions between subcomponents of risk scores and sex in lung function and height. Since interactions for complex traits at the single genetic variant level were rarely detected, even in recent large-scale analyses, we hypothesize that our approach will be an important tool for the identification of genetic interactions and the underlying mechanisms.

Fawcett et al. performed a genome-wide genotype-by-sex interaction analysis of lung function in the UK Biobank, using more than 300,000 samples [40]. Although they found a small number of five genome-wide significant interactions, only one interaction signal was replicated in an independent study. In a different study, Bernabeu et al. utilized the UK Biobank to analyze genotype by sex interactions for 530 complex traits. This analysis revealed several genome-wide significant interaction findings, but heritability and genetic correlation analyses suggest that substantial proportions of the sex-differential genetic architecture are yet to be discovered and identified [42]. Our results are in line with these findings since our interaction signals are not driven by strong effects at the single variant level but aggregations across sets of variants. Furthermore, recent work by Zhu et al. hypothesizes and demonstrates that so-called amplification is a common mode of genotype-by-sex interactions [43]. Amplification describes the setting of shared genetic variants, with the same direction of effect, but different magnitudes. This is exactly what we observed in our UK Biobank sex interaction analysis of lung function and human height. However, we note that analyses based on independent datasets are required to further replicate, validate, and disentangle these potential gene-by-sex interactions.

Our approach has the following limitations. A significant interaction p-value of the aggregated score does not imply that all included pairs of genetic variants/environmental factors necessarily truly contribute to the interaction signal. Another limitation is that the set of genetic variants needs to be pre-selected and, as described in the Methods section, the size *m* is assumed to be of moderate magnitude in the range of 10^2^−10^3^ (assuming typical GWAS sample sizes). This restriction is related to the required assumption that the main effects and transformations can be estimated accurate enough given the sample size. The computational burden, depending on the specific implementation of the screening strategy, also increases with *m*. Therefore, an important question is how to pre-select the set of genetic variants for RITSS testing. It is possible to integrate an external screening approach. For a quantitative trait, one can screen for potential interaction signals based on marginal effects or variance-effects [10,14]. Based on the results of such a screening, one can choose sets of genetic variants for RITSS testing and introduce weighted hypotheses reflecting the results of this external screening approach. It is important to note that this screening should not be confused with the screening and testing steps within the RITSS framework. In the UK Biobank application, we restricted the input to previously reported GWAS findings for the respective trait, which can be interpreted as a special case of this prior external screening. However, interaction effects do not need to be accompanied by marginal effects and therefore our analysis provides only a limited insight.

In general, the interpretation of significant RITSS p-values requires careful consideration. RITSS aims to identify deviations from the null hypothesis model $E[Y_{i}|X_{i},Z_{i},E_{i}]=\mu (E_{i},Z_{i})+\sum_{j=1}^{m}\pi_{0j}(Z_{i})X_{ij}$, represented as significant interaction scores *U*_*i*_ = ∑_*j*_∑_*l*_*π*_*jl*_*X*_*ij*_*E*_*il*_. However, a significant contribution by *U*_*i*_ does not imply interaction between *E*_*i*_ and *X*_*i*_ without assumptions regarding no unmeasured confounding/omitted variables and the sampling design [44,45]. Moreover, measurement of environmental factors is often subject to noise or even bias. This can result in false positive interaction findings since RITSS assumes that environmental factors are measured without error. Furthermore, due to the computational burden, our type 1 error simulations are based on 10,000 replicates only and, therefore, the validity at small significance levels cannot be empirically investigated. Nevertheless, we emphasize that the RITSS approach is supported by theoretical results describing the asymptotic normality of the test statistic. The simulation results demonstrated the concordance with these theoretical approximations. Finally, due to the application of sample splitting and several independent estimation steps, RITSS requires datasets with sample sizes that are in the order of recent GWAS publications (30,000 samples or more).

Future directions include more detailed follow-up analyses of the observed gene-by-sex interactions, the incorporation of other screening techniques, such as, for example, LASSO [46] or RaSE [47], and the improvement of the computational efficiency. We plan to extend RITSS to incorporate dichotomous traits and implement these extensions in the R package *RITSS*. We note that RITSS can also be used to analyze gene-gene-interactions and interaction models can be based on more general, non-linear approaches. In addition, RITSS can be applied to other omics data layers, such as DNA methylation, transcriptomics, or metabolomics. The R package *RITSS* as well as the code for the simulation studies is available at https://github.com/julianhecker/RITSS.

## Supporting information

S1 Appendix ACE algorithm and RITSS main effects, Screening strategies, Implementation of GAMsv, Simulation details, Additional simulation study results, and UK Biobank data. This appendix contains additional information regarding the ACE algorithm and RITSS main effects, the screening strategies, the implementation of GAMsv, simulation details, additional simulation study results, and the UK Biobank data.
(PDF)

S2 Appendix Additional information regarding genetic variants tested in the UK Biobank analysis. These tables contain all genetic variants as well as the *m*_4_/*m*_3_ information for the sex-interaction analysis in the UK Biobank.
(XLSX)

S1 Fig Density plots for standardized residuals for all four traits in the analysis. FEV_1_: forced expiratory volume in 1 second, FVC: forced vital capacity.
(TIF)
