# Supplementary material for: A robust and adaptive framework for interaction testing in quantitative traits between multiple genetic loci and exposure variables
Source: PLoS Genet. 2022 Nov 16;18(11):e1010464. doi: 10.1371/journal.pgen.1010464 (PMC9668174; doi:10.1371/journal.pgen.1010464)
Supplement: S1 Appendix — This appendix contains additional information regarding the ACE algorithm and RITSS main effects, the screening strategies, the implementation of GAMsv, simulation details, additional simulation study results, and the UK Biobank data. (PDF) [file pgen.1010464.s001.pdf]

Contents

ACE algorithm and RITSS main effects 1
Screening strategies 2
Implementation of GAMsv 4
Simulation details 4
Additional simulation study results 8
UK Biobank data 10
References 11

ACE algorithm and RITSS main effects

ACE algorithm

The input for the ACE algorithm [1,2] consists of the score U_i, genetic data X_i, environmental factors E_i, and the covariates Z_i. The goal is to derive a transformation of U_i that is orthogonal to functions $\mu(E_i, Z_i)$ (i.e., $\boldsymbol{E}[U_i'|E_i, Z_i] = 0$) and orthogonal to the genetic main effects $\sum_{j=1}^{m} \pi_{0j}(Z_i) X_{ij}$.

Starting from an input score of the form $U_i = \sum_j \sum_l \pi_{jl} X_{ij} E_{il}$, we

1. estimate $\boldsymbol{E}[U_i|X_i, Z_i] = \sum_l \boldsymbol{E}[E_{il}|X_i, Z_i] \sum_j \pi_{jl} X_{ij}$ using a flexible model and replace U_i by the corresponding residuals.
2. Use a statistical/machine learning approach to estimate $\boldsymbol{E}[U_i|E_i, Z_i]$ and replace U_i by the corresponding residuals.
3. Use a statistical/machine learning approach to estimate $\boldsymbol{E}[U_i|X_i, Z_i]$ and replace U_i by the corresponding residuals.
4. Repeat steps 2.) and 3.) until a suitable convergence criterion is reached [3].

For step 2.), we applied generalized additive models (GAMs) [4] using the *bam* function in the *mgcv* R package [5–7]. For these models, we used cubic regression splines with $k = 3$ for each factor separately, and default options otherwise. For step 1.) and 3.), we used a LASSO-based linear model based on X_i and Z_i, including interaction terms; the tuning parameter was chosen using cross validation. In practice, 2-3 iterations of steps 2.) + 3.) are often sufficient.

RITSS main effects implementation in the simulation studies and the UK Biobank application

The main effect models in RITSS were implemented using the *bam* function in the *mgcv* R package [5–7]. The genetic main effects were realized as non-smooth standard covariates (additive genetic model), assuming no interactions with Z_i. We implemented an option to include these interactions. The interaction scores U_{is} were fitted as standard covariates. The environmental main effect $\mu(E_i, Z_i)$ was realized using cubic regression splines with $k = 3$ for each factor separately, otherwise default settings.

Screening strategies

Screening strategies 1: interaction between single environmental factor and subcomponent of genetic risk score

Using I_1^{c1} data, we first fit the model $Y_i = \mu_0 + E_i^T \mu_1 + Z_i^T \mu_2 + X_i^T \pi_0 + \varepsilon_i$. Then, we construct the matrix $X_{ij}^{Wt} = \hat{\pi}_{0j} X_{ij} E_{it}$ where t is the index of the environmental factor of interest. The matrix X_{ij}^{Wt} describes genotype-environment products weighted by the corresponding estimated genetic main effect $\hat{\pi}_{0j}$. Next, we regress out X_i, E_i, and Z_i from Y_i and X_{ij}^{Wt} (for each variant j separately) and denote the resulting residuals by Y_i^r and X_{ij}^{Wtr}. The variant-wise variances of $X_{.j}^{Wtr}$, denoted by v_j, differ typically substantially due to the different estimated genetic main

effects and minor allele frequencies. We keep only variants whose variance is larger than $\frac{1}{m} * \sum_j v_j$ and denote the resulting number of variants by S_{max}.

Based on these objects, we split I_1^{c1} randomly in two equally sized parts and perform approximate best subset selection [8] in each part with different subset sizes, where the corresponding other part is used as testing data. The best subset here corresponds to the best subset of variants, and we do not utilize the effect estimates per variant inferred in the best subset regression. Instead, we sum X_{ij}^{Wtr} over the corresponding variants, describing the genetic risk score multiplied by the environmental factor. The size of the subsets is increased between 10 and S_{max}, in steps of size 5. We select the best subsets in both parts of the data in terms of the association with Y_i^r in the test data and check the overlap between these subsets to create two different scores. Denote by J_1 the set of overlapping variants (contained in both best subsets), and by J_2 the set of non-overlapping variants that were only in one best subset. The first score is then defined by $U_{i1} = \sum_{j \epsilon J_1} X_{ij}^{Wt}$, and the second by $U_{i2} = \sum_{j \epsilon J_2} X_{ij}^{Wt}$.

Screening strategies 2: aggregated single environmental factor interactions based on single variant testing

Based on the model $Y_i = \mu_0 + E_i^T \mu_1 + Z_i^T \mu_2 + X_{ij}\pi_{0j} + E_{it}X_{ij}\pi_{jt} + \varepsilon_i$ for each genetic variant j in step b.), we select all variants where the FDR q-value for π_{jt} is below 0.05 in the first score, and all variants where the FDR q-value for π_{jt} is between 0.05 and 0.10 in the second score. The corresponding estimated interaction effects $\hat{\pi}_{jt}$ are used to construct the corresponding interaction score. If there are no variants with FDR q-value below 0.1, we keep the variants with the smallest interaction p-value and construct only one score. If there is no variant with FDR q-

value below 0.05, but below 0.1, we construct only one score with all variants with FDR q-value below 0.1.

Implementation of GAMsv

The alternative approach GAMsv was implemented using the *bam* function in the *mgcv* R package [5–7]. The genetic main effect of a single variant X_{ij} was modeled as a non-smooth standard covariate (additive genetic model), the same applies to the standard interaction term $X_{ij}E_{it}$. The environmental main effect $\mu(E_i, Z_i)$ was realized using cubic regression splines with $k = 3$ for each factor separately; otherwise default settings were applied.

Simulation details

For all simulations, we set $n = 30{,}000$, $m = 100$, $d = 5$, and $p = 2$.

Type 1 error simulations

The covariates Z_{ij} are simulated independently based on a uniform distribution $Z_{ij} \sim Unif(a, b), j = 1, \ldots, p$. They are used to simulate population stratification and can be interpreted as principal components related information (although not orthogonal). The parameters a and b are set to $a = 0.0$ and $b = 0.1$. The genotypes X_{ij} are generated based on a binomial distribution with $X_{ij} \sim Binom(2, p = 0.3 + Z_{iv}), j = 1, \ldots, m$. The index $v = v(j) \epsilon \{1, \ldots, p\}$ is chosen based on a varying sequence that depends on m and p, to ensure approximate equal coverage of all components of Z_i. The environmental factors E_{ij} follow $E_{ij} = X_i^T \beta_{EXj} + Z_i^T \beta_{EZj} + W_{ij}, j = 1, \ldots, d$, with $W_{ij} \sim N(0,1)$ independent. The vectors β_{EXj} and β_{EZj} control gene-environment correlations and population-specific differences in the environment.

The residual error ε_i is simulated by $\varepsilon_i = b_i * (1 + |\beta_{\varepsilon E}| * E_{i1})$, where $b_i \sim N(0,1)$ independent in the case of normal errors. In the scenario of non-normal errors, b_i is sampled from the mean-centered and standardized lung function ratio in the UK Biobank [9]. The parameter $\beta_{\varepsilon E}$ controls the presence of heteroscedastic errors. The phenotype Y_i is computed by:

$$Y_i = X_i^T \pi_0 + \mu(E_{i1}, \beta_{E1}) + E_{i2}\beta_{E2} + \cdots + E_{id}\beta_{Ed} + Z_i^T \beta_Z + \varepsilon_i$$

The effects $\pi_0, \beta_E, \beta_Z, \beta_{EZj}, \beta_{EXj}$, and $\beta_{\varepsilon E}$ are generated as follows:

$$\beta_Z \sim N(0, \sigma_Z^2 I_p),\ \beta_{\varepsilon E} \sim N(0, \sigma_{\varepsilon E}^2),$$

$\beta_E = |\alpha_E|$, where $\alpha_E \sim N(0, \sigma_E^2 I_d)$ (component-wide absolute value),

$\pi_0 = B\pi_0'$, where $\pi'_0 \sim N(0, \sigma_X^2 I_m)$ and $B \sim Bernoulli(0.5, m)$ independent (component-wise product, $Bernoulli(x, k)$ denotes a k dimensional vector with identically and independently distributed Bernoulli components with success probability x), and

$\beta_{EXj} = B_j|\alpha_{EXj}|$ (component-wise absolute value) where $\alpha_{EXj} \sim N(0, \sigma_{EX}^2 I_m)$, $j = 1, \ldots, d$ and $B_j \sim Bernoulli(0.5, m)$ independent (component-wise product) as well as

$\beta_{EZj} = B'_j \alpha_{EZj}$ where $\alpha_{EZj} \sim N(0, \sigma_{EZ}^2 I_p)$, $j = 1, \ldots, d$ and $B'_j \sim Bernoulli(0.5, p)$ independent (component-wise product). The parameters $\pi_0, \beta_E, \beta_Z, \beta_{EZj}, \beta_{EXj}$, and $\beta_{\varepsilon E}$ are freshly drawn in each replication of the simulation study. We set $\sigma_X^2 = 0.1^2, \sigma_{EZ}^2 = 0.1^2$, and $\sigma_Z^2 = 0.1^2$. In the following Table A, we report scenario-dependent parameter values/implementations.

scenario	σ_E^2	σ_{EX}^2	$\sigma_{\varepsilon E}^2$	$\mu(E_{i1}, \beta_{E1})$	b_i
1	$\sigma_E^2 = 0.1^2$	$\sigma_{EX}^2 = 0.0^2$	$\sigma_{\varepsilon E}^2 = 0.0^2$	$\beta_{E1}E_{i1}$	$b_i \sim N(0,1)$
2	$\sigma_E^2 = 0.3^2$	$\sigma_{EX}^2 = 0.0^2$	$\sigma_{\varepsilon E}^2 = 0.0^2$	$\beta_{E1}E_{i1}^2$	$b_i \sim N(0,1)$
3	$\sigma_E^2 = 0.1^2$	$\sigma_{EX}^2 = 0.0^2$	$\sigma_{\varepsilon E}^2 = 0.5^2$	$\beta_{E1}E_{i1}^2$	$b_i \sim \frac{FEV_1}{FVC}$ UK Biobank, standardized
4	$\sigma_E^2 = 0.1^2$	$\sigma_{EX}^2 = 0.1^2$	$\sigma_{\varepsilon E}^2 = 0.0^2$	$\beta_{E1}E_{i1}^2$	$b_i \sim N(0,1)$
5	$\sigma_E^2 = 0.1^2$	$\sigma_{EX}^2 = 0.1^2$	$\sigma_{\varepsilon E}^2 = 0.2^2$	$\beta_{E1}E_{i1}^2$	$b_i \sim \frac{FEV_1}{FVC}$ UK Biobank, standardized

Table A: Scenario-dependent parameter values for the simulation studies.

The SELECT:yes/no scenarios were implemented as follows:

- SELECT:no: all variants are included.
- SELECT:yes, if the number of variants with association p-value $p < 5 * 10^{-8}$ (based on all n samples) is between 30% and 50% of m, keep only these variants. If not: keep the $\frac{m}{2}$ variants with the smallest association p-values.

Power simulations

For the power simulations, we used scenario 1 from the type 1 error simulations with SELECT:no and added a gene-environment interaction term to the model. The full model is given by

$$Y_i = E_i^T \beta_E + Z_i^T \beta_Z + X_i^T \pi_0 + (E_{i1} X_i)^T \pi_1 + \varepsilon_i$$

where $E_{i1}X_i$ is the suitable component-wise product. We modeled π_1 given π_0 using $\pi_{1j} = \pi_{0j} * |d_j| * b_j$, with $d_j \sim N(\mu_{XE}, \sigma_{XE}^2)$ and $b_j \sim Bernoulli(p_{XE})$ for $j = 1, \ldots, m$. The genetic main effects

π_0 are modeled as in the type 1 error simulations. In the power simulations, we considered all combinations of $\mu_{XE} = 0.05, 0.1$, $\sigma_{XE}^2 = 0.01^2, 0.05^2, 0.1^2$, and $p_{XE} = 0.05, 0.1, 0.2, 0.3, 0.4, 0.5$.

Additional simulation study results

scenario	K/c	RITSS1 KS test	RITSS2 KS test	RITSS1 #significant	RITSS2 #significant	D1 #significant	D2 #significant
1	$3/(0.5, 0.25, 0.25)$	1.0000	1.0000	0	0	71	50
2	$3/(0.5, 0.25, 0.25)$	1.0000	0.6535	0	1	69	57
3	$3/(0.5, 0.25, 0.25)$	1.0000	1.0000	0	0	63	50
4	$3/(0.5, 0.25, 0.25)$	1.0000	0.0027	1	2	518	653
5	$3/(0.5, 0.25, 0.25)$	0.0046	0.1108	0	0	485	576
1	$3/(1/3, 1/3, 1/3)$	1.0000	1.0000	0	0	48	32
2	$3/(1/3, 1/3, 1/3)$	1.0000	1.0000	0	0	52	31
3	$3/(1/3, 1/3, 1/3)$	1.0000	1.0000	0	1	62	20
4	$3/(1/3, 1/3, 1/3)$	1.0000	0.1480	0	1	477	578
5	$3/(1/3, 1/3, 1/3)$	1.0000	1.0000	0	0	427	500
1	$4/(0.5, 0.25, 0.25)$	1.0000	1.0000	0	1	49	43
2	$4/(0.5, 0.25, 0.25)$	1.0000	0.5193	0	1	46	38
3	$4/(0.5, 0.25, 0.25)$	1.0000	1.0000	0	0	39	31
4	$4/(0.5, 0.25, 0.25)$	1.0000	0.2154	0	0	489	630
5	$4/(0.5, 0.25, 0.25)$	1.0000	0.1278	0	0	447	565
1	$4/(1/3, 1/3, 1/3)$	1.0000	1.0000	0	0	57	20
2	$4/(1/3, 1/3, 1/3)$	1.0000	1.0000	0	0	21	27
3	$4/(1/3, 1/3, 1/3)$	1.0000	1.0000	0	0	40	27
4	$4/(1/3, 1/3, 1/3)$	1.0000	1.0000	0	0	431	543
5	$4/(1/3, 1/3, 1/3)$	1.0000	0.2522	0	0	404	464

Table B: Type 1 error simulation results based on 1,000 replicates. K/c denotes the configuration of K and the splitting fraction c. RITSS1 KS test and RITSS2 KS test report the p-value of the Kolmogorov-Smirnov test, Bonferroni corrected for 40 tests (5 x 2 x 4). This test compared the p-values of RITSS1 and RITSS2 with a standard uniform distribution. The last columns report the number of significant (Bonferroni) interaction p-values, that is, the number of p-values with $p < \frac{0.05}{1000}$.

σ_{XE}	p_{XE}	K/c	RITSS1	RITSS2
0.01	0.05	$3/(0.5, 0.25, 0.25)$	0.0240	0.0140
0.01	0.05	$3/(1/3, 1/3, 1/3)$	0.0190	0.0070
0.01	0.05	$4/(0.5, 0.25, 0.25)$	0.0180	0.0150
0.01	0.05	$4/(1/3, 1/3, 1/3)$	0.0130	0.0080
0.01	0.2	$3/(0.5, 0.25, 0.25)$	0.1470	0.0430
0.01	0.2	$3/(1/3, 1/3, 1/3)$	0.1320	0.0240
0.01	0.2	$4/(0.5, 0.25, 0.25)$	0.1470	0.0340
0.01	0.2	$4/(1/3, 1/3, 1/3)$	0.1340	0.0230
0.01	0.5	$3/(0.5, 0.25, 0.25)$	0.6730	0.0940
0.01	0.5	$3/(1/3, 1/3, 1/3)$	0.6350	0.0620
0.01	0.5	$4/(0.5, 0.25, 0.25)$	0.6630	0.0990
0.01	0.5	$4/(1/3, 1/3, 1/3)$	0.6430	0.0550
0.1	0.05	$3/(0.5, 0.25, 0.25)$	0.0370	0.0550
0.1	0.05	$3/(1/3, 1/3, 1/3)$	0.0310	0.0360
0.1	0.05	$4/(0.5, 0.25, 0.25)$	0.0470	0.0620
0.1	0.05	$4/(1/3, 1/3, 1/3)$	0.0420	0.0390
0.1	0.2	$3/(0.5, 0.25, 0.25)$	0.2550	0.2240
0.1	0.2	$3/(1/3, 1/3, 1/3)$	0.2280	0.1600
0.1	0.2	$4/(0.5, 0.25, 0.25)$	0.2700	0.2420
0.1	0.2	$4/(1/3, 1/3, 1/3)$	0.2480	0.1520
0.1	0.5	$3/(0.5, 0.25, 0.25)$	0.7710	0.4880
0.1	0.5	$3/(1/3, 1/3, 1/3)$	0.7330	0.3580
0.1	0.5	$4/(0.5, 0.25, 0.25)$	0.7750	0.5110
0.1	0.5	$4/(1/3, 1/3, 1/3)$	0.7700	0.3680

Table C: Power simulation results based on 1,000 replicates and $\mu_{XE} = 0.1$. K/c denotes the configuration of K and the splitting fraction c. RITSS1 and RITSS2 report the empirical power at a significance threshold of $\alpha = 0.005$.

UK Biobank data

Study population

Our data analysis utilized participants from the UK Biobank [9]. We selected only participants of European ancestry, where ancestry was derived based on a combination of self-reported ethnicity and k-means clustering of principal components of genetic ancestry, as previously described [10]. Besides standard filters for the UK Biobank, quality control included the exclusion of related pairs, and excluding participants whose spirometry data did not meet quality control standards [10,11] or that had missing phenotype/covariate data (see below). Overall, we kept 254,033 participants for the analysis.

Phenotype and covariate data

We incorporated lung function data as measured by forced expiratory volume in 1 second (FEV_1), forced vital capacity (FVC), and the ratio FEV_1/FVC. We also extracted age, sex, standing height, smoking exposure variables, genotyping array, and the first ten principal components of genetic ancestry. Smoking exposure was based on self-reports and included the variables pack-years of smoking (P-Y-S) and ever- versus never-smoking status (E-S). 'Ever-smokers' included individuals reporting current smoking, smoking most days, smoking occasionally, or former smoking. 'Never smokers' included those who smoked less than 100 cigarettes in their lifetime. Standing height is referred to as height in the main text.

Genetic data

Genotyping and imputation for the UK Biobank were performed as described in the corresponding publications [9,10]; this genetic data was directly available for our analyses. For each of the four phenotypes FEV_1, FVC, FEV_1/FVC, and height, we downloaded all reported

genetic associations from the GWAS catalog [12] (February-August, 2021) (EFO_0004314, EFO_0004312, EFO_0004713, and EFO_0004339, respectively). We extracted the corresponding genetic variants with a minor allele frequency above 1% (estimated in the analysis dataset) and performed LD pruning *(indep-pairwise* command with parameters 500, 50, and 0.2) using PLINK2 (version v2.00a2.3LM) [13] to exclude genetic variants that are in strong LD. We also excluded multi-allelic variants. Our analyses are based on expected minor allele count information, as computed by PLINK2. The final numbers of variants for the analysis of each respective phenotype are described in Table 1 of the main text.

References

1. Breiman L, Friedman JH. Estimating Optimal Transformations for Multiple Regression and Correlation: Rejoinder. J Am Stat Assoc. 1985;80: 614–619. doi:10.2307/2288477

2. Vansteelandt S, VanderWeele TJ, Robins JM. Multiply robust inference for statistical interactions. J Am Stat Assoc. 2008;103: 1693–1704. doi:10.1198/016214508000001084

3. Vansteelandt S, Dukes O. Assumption-lean inference for generalised linear model parameters. 2020 [cited 26 Oct 2022]. doi:10.48550/arXiv.2006.08402

4. Hastie T, Tibshirani R. Generalized Additive Models. Stat Sci. 1986;1: 297–310. doi:10.1214/ss/1177013604

5. Wood SN. Stable and Efficient Multiple Smoothing Parameter Estimation for Generalized Additive Models. J Am Stat Assoc. 2004;99: 673–686. doi:10.1198/016214504000000980

6. Wood SN. Fast stable restricted maximum likelihood and marginal likelihood estimation of semiparametric generalized linear models. J R Stat Soc Ser B Stat Methodol. 2011;73: 3–36. doi:10.1111/j.1467-9868.2010.00749.x

7. Wood SN. Generalized Additive Models: An Introduction with R. 2nd ed. New York: Chapman and Hall/CRC; 2017. doi:10.1201/9781315370279

8. Bertsimas D, King A, Mazumder R. Best subset selection via a modern optimization lens. Ann Stat. 2016;44: 813–852. doi:10.1214/15-AOS1388

9. Bycroft C, Freeman C, Petkova D, Band G, Elliott LT, Sharp K, et al. The UK Biobank resource with deep phenotyping and genomic data. Nature. 2018;562: 203–209. doi:10.1038/s41586-018-0579-z

10. Shrine N, Guyatt AL, Erzurumluoglu AM, Jackson VE, Hobbs BD, Melbourne CA, et al. New genetic signals for lung function highlight pathways and chronic obstructive pulmonary disease associations across multiple ancestries. Nat Genet. 2019;51: 481–493. doi:10.1038/s41588-018-0321-7

11. Kim W, Moll M, Qiao D, Hobbs BD, Shrine N, Sakornsakolpat P, et al. Interaction of Cigarette Smoking and Polygenic Risk Score on Reduced Lung Function. JAMA Netw Open. 2021;4: e2139525. doi:10.1001/jamanetworkopen.2021.39525

12. MacArthur J, Bowler E, Cerezo M, Gil L, Hall P, Hastings E, et al. The new NHGRI-EBI Catalog of published genome-wide association studies (GWAS Catalog). Nucleic Acids Res. 2017;45: D896–D901. doi:10.1093/nar/gkw1133

13. Chang CC, Chow CC, Tellier LC, Vattikuti S, Purcell SM, Lee JJ. Second-generation PLINK: rising to the challenge of larger and richer datasets. GigaScience. 2015;4: 7. doi:10.1186/s13742-015-0047-8
