# Supplementary figures and images for: A robust and adaptive framework for interaction testing in quantitative traits between multiple genetic loci and exposure variables
Source: PLoS Genet. 2022 Nov 16;18(11):e1010464. doi: 10.1371/journal.pgen.1010464 (PMC9668174; doi:10.1371/journal.pgen.1010464)

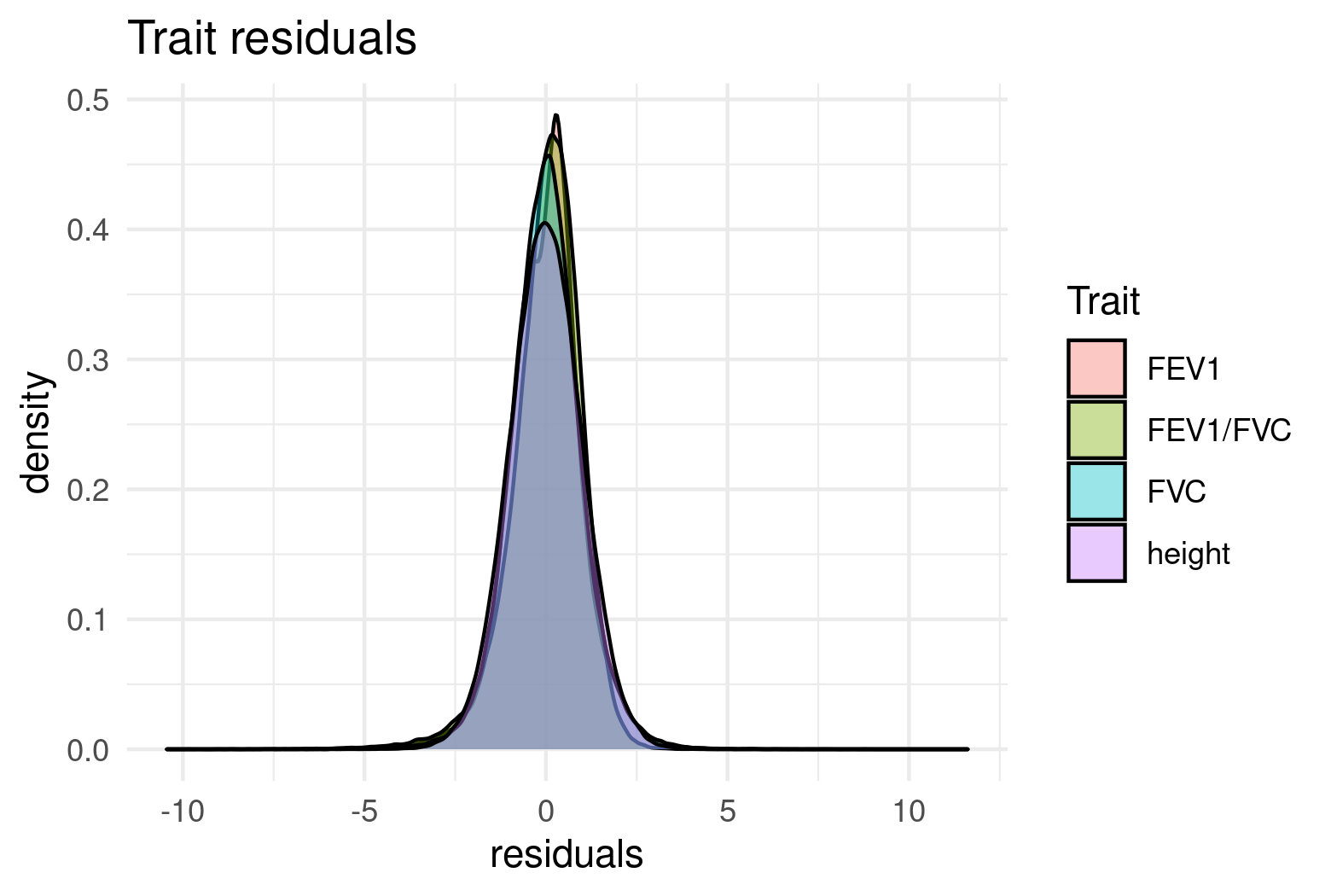

Supplement: S1 Fig — FEV1: forced expiratory volume in 1 second, FVC: forced vital capacity. (TIF) [file pgen.1010464.s003.tif]
